# The influence of marital status on survival in patients with oral tongue squamous cell carcinoma

**DOI:** 10.18632/oncotarget.18538

**Published:** 2017-06-17

**Authors:** Wei Sun, Zeting Qiu, Wulin Tan, Zhongqi Liu, Zhongxing Wang, Wenqi Huang, Minghui Cao

**Affiliations:** ^1^ Department of Anesthesiology, Sun Yat-sen Memorial Hospital, Sun Yat-sen University, Guangzhou, Guangdong, China; ^2^ Department of Anesthesiology, The First Affiliated Hospital of Sun Yat-sen University, Guangzhou, Guangdong, China

**Keywords:** oral tongue squamous cell carcinoma, marital status, SEER, survival analysis, biopsychosocial medical model

## Abstract

Marital status was found to be an independent prognostic factor for survival in several cancers. However related researches of oral tongue squamous cell carcinoma (OTSCC) are still rare. We explored the Surveillance, Epidemiology, and End Results (SEER) program and finally identified 14,194 patients with OTSCC. Kaplan-Meier analysis and multivariate Cox regression models were used to distinguish risk factors for overall survival (OS) and tumor cause-specific survival (TCSS). Widowed patients had the highest percentage of female, highest average ages and more prevalence with localized SEER Stage significantly, while patients in the single group were younger than other groups. After univariate analysis and multivariate analysis, marital status was demonstrated to be an independent prognostic factor of OS and TCSS. Married patients showed better 5-year OS (65.6%) and 5-year TCSS (89.9%) than other patients. Subgroup survival analysis according to AJCC TNM stage and SEER stage showed that the widowed patients demonstrated worst OS and TCSS compared to other groups. Marital status was an important prognostic factor for survival in patients with OTSCC. Widowed patients exhibited with the highest risk of death compared with other groups.

## INTRODUCTION

Cancers of lip and oral cavity affected about 300,373 new cases and killed 145,353 people all over the world in 2012 based on GLOBOCAN estimates [1]. Oral tongue squamous cell carcinoma (OTSCC) is oral cavity squamous cell carcinoma (OCSCC) originating from the tongue. Etiologically, tobacco smoking and alcohol use is two principal risk factors of OCSCC [2]. Besides tobacco and alcohol, infection with human papilloma virus (HPV) especially HPV 16 is strongly related with occurrence of tongue cancer [3]. OCSCC including OTSCC can be classified into stage I-IV according to the tumor node metastases (TNM) staging system of the American Joint Committee on Cancer (AJCC) [4]. For the AJCC stage I and II early OTSCCs the five-year survival rates was 67% and 51%, while for stage III and IV advanced OTSCCs the five-year disease-specific survival rates was 39% and 27% [5, 6]. Nowadays the notions of health and disease have more and more emphasized the position of social and psychological factors in disease development, which is also called biopsychosocial medical model [7, 8]. Nevertheless almost all the clinical studies of tongue cancer concentrated on the importance of clinical factors [9, 10], while few studies focused on the roles of social and psychological factors.

Stable marital status represents a solid marriage, which could lead to a positive social support and psychological state, and finally improves cancer survival [11]. It has been demonstrated that marital status acts as an independent prognostic factor for survival in several cancers, such as breast cancer, gastric cancer and pancreatic cancer [12–14]. However there is no study analyzing the influence of marital status on prognosis in oral tongue squamous cell carcinoma yet. Given that oral tongue squamous cell carcinoma has become a horrible threat worldwide, it is meaningful to investigate the relationship between marital status and tongue cancer survival.

The Surveillance, Epidemiology, and End Results (SEER) program are composed of 18 cancer registries covering approximately 30% of the population in the United States [15, 16]. It provides complete patient data including demographic information, clinical records and follow-up data updated annually by the National Center for Health Statistics. In this study, we took use of SEER data to analyze the influence of marital status on survival in patients with oral tongue squamous cell carcinoma.

## RESULTS

### Patient baseline characteristics

With the inclusion criteria, we initially included 27,871 patients from the SEER database. Then we excluded seven patients who were less than 18 years, 7,711 patients with incomplete clinical information, 2,242 patients with unknown demographic information and 3,717 patients with unknown cause of death or unknown survival month. Finally we identified 14,194 eligible patients with oral tongue squamous cell carcinoma, including 10,137 (71.4%) male and 4,057 (28.6%) female patients. Of these, 8,298 (58.5%) patients were married, 2,705 (19.1%) were single, 2,001 (14.1%) were divorced/separated and 1,190 (8.4%) were widowed respectively. Baseline demographic information and clinical record of all the included patients were showed in Table 1. There were significant differences in gender, age, race, marital status, grade, AJCC TNM stage, SEER stage, therapy of surgery and therapy of radiation among different groups. Single patients were younger (54.9 ± 12.8 years old), had the lowest proportion (79.4%) of white and the highest proportion (16.1%) of black when compared with married, divorced/separated and widowed patients. Patients in the widowed group had the highest percentage (67.2%) of female, highest average ages (74.0 ± 11.1 years old) and more prevalence (39.0%) with localized SEER Stage significantly. Widowed patients also had a larger proportion (59.4%) of receiving surgery and a smaller proportion (38.6%) of receiving radiation compared with others.

**Table 1:** Baseline characteristic of patients with oral tongue squamous cell carcinoma in SEER database

Characteristic (%)		Total	Married	Single	Divorced/Separated	Widowed	*P* value
		(%)	(%)	(%)	(%)		
		14194 (100.0)	8298 (58.5)	2705 (19.1)	2001 (14.1)	1190 (8.4)	
Gender							< 0.001
	Male	10137 (71.4)	6294 (75.8)	1994 (73.7)	1459 (72.9)	390 (32.8)	
	Female	4057 (28.6)	2004 (24.2)	711 (26.3)	542 (27.1)	800 (67.2)	
Age							< 0.001
		60.2 ± 12.5	60.0 ± 12.1	54.9 ± 12.8	60.1 ± 10.1	74.0 ± 11.1	
Race							< 0.001
	White	12218 (86.1)	7343 (88.5)	2149 (79.4)	1744 (87.2)	982 (82.5)	
	Black	1059 (7.5)	328 (4.0)	436 (16.1)	180 (9.0)	115 (9.7)	
	Others	917 (6.5)	627 (7.6)	120 (4.4)	77 (3.8)	93 (7.8)	
Grade							< 0.001
	I	2108 (14.9)	1247 (15.0)	388 (14.3)	264 (13.2)	209 (17.6)	
	II	6999 (49.3)	3953 (47.6)	1420 (52.5)	995 (49.7)	631 (53.0)	
	III	4967 (35.0)	3018 (36.4)	882 (32.6)	720 (36.0)	347 (29.2)	
	IV	120 (0.8)	80 (1.0)	15 (0.6)	22 (1.1)	3 (0.3)	
AJCC stage							< 0.001
	I	3183 (22.4)	2035 (24.5)	520 (19.2)	313 (15.6)	315 (26.5)	
	II	1672 (11.8)	942 (11.4)	294 (10.9)	234 (11.7)	202 (17.0)	
	III	2392 (16.9)	1395 (16.8)	471 (17.4)	319 (15.9)	207 (17.4)	
	IV	6947 (48.9)	3926 (47.3)	1420 (52.5)	1135 (56.7)	466 (39.2)	
SEER stage							< 0.001
	Localized	4345 (30.6)	2678 (32.3)	742 (27.4)	461 (23.0)	464 (39.0)	
	Regional	7316 (51.5)	4376 (52.7)	1359 (50.2)	1072 (53.6)	509 (42.8)	
	Distant	2533 (17.8)	1244 (15.0)	604 (22.3)	468 (23.4)	217 (18.2)	
Surgery							< 0.001
	Yes	8000 (56.4)	4821 (58.1)	1491 (55.1)	981 (49.0)	707 (59.4)	
	No	6194 (43.6)	3477 (41.9)	1214 (44.9)	1020 (51.0)	483 (40.6)	
Radiation							< 0.001
	Yes	6021 (42.4)	3411 (41.1)	1165 (43.1)	986 (49.3)	459 (38.6)	
	No	8173 (57.6)	4887 (58.9)	1540 (56.9)	1015 (50.7)	731 (61.4)	

Notes: AJCC, the American Joint Committee on Cancer; SEER, the Surveillance, Epidemiology and End Results.

### The influence of marital status on overall survival (OS)

**Figure 1:**
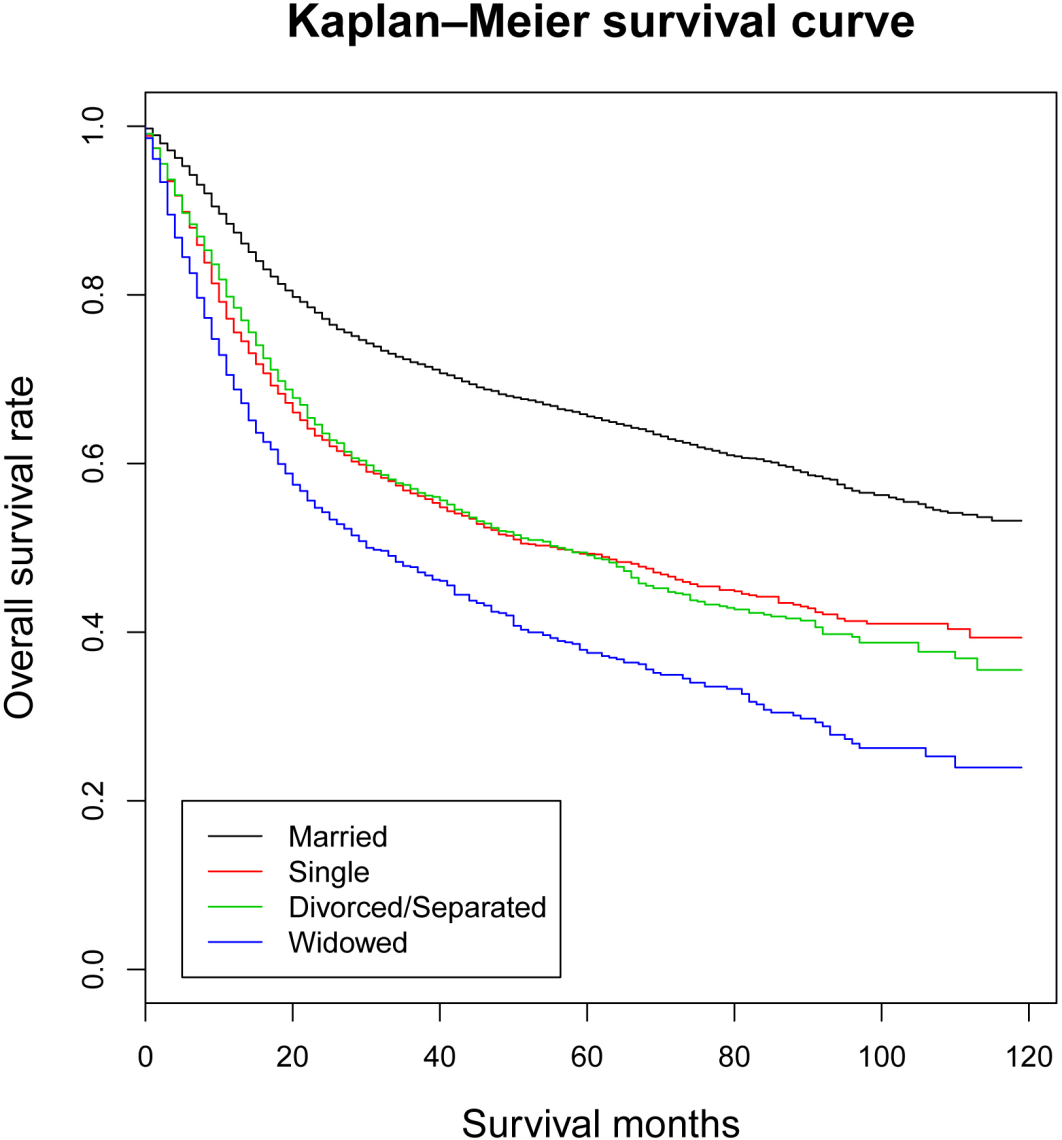
Kaplan-Meier survival curves: the overall survival in patients with oral tongue squamous cell carcinoma according to marital status χ^2^ = 569, *P <* 0.001.

Univariate analysis (Kaplan-Meier analysis) and multivariate analysis (multivariate Cox regression analysis) were used to evaluate the overall survival (OS) of oral tongue squamous cell carcinoma patients (Table 2 and Supplementary Table 1). The 5-year OS rate was 65.6% in the married group, 49.3% in the single group, 49.1% in the divorced/separated group and 37.5% in the widowed group respectively. Univariate analysis discovered age (*P* < 0.001), race (*P <* 0.001), marital status (*P <* 0.001), grade (*P <* 0.001), AJCC TNM stage (*P <* 0.001), SEER stage (*P <* 0.001), therapy of surgery (*P <* 0.001) and therapy of radiation (*P <* 0.001) as significant factors associated with OS. After including and adjusting all these significant variables in the multivariate analysis, all the factors containing age (*P <* 0.001), race (*P <* 0.001), marital status (*P* < 0.001), grade (*P <* 0.001), AJCC TNM stage (*P <* 0.001), SEER stage (*P <* 0.001), therapy of surgery (*P <* 0.001) and therapy of radiation (*P <* 0.001) remained as independent prognostic factors. When it came to marital status, married patients had better OS than other OTSCC patients (Single, HR [hazard ratio] 1.645, 95% CI [confidence interval] 1.531–1.767, *P <* 0.001; Divorced/Separated, HR 1.517, 95% CI 1.404–1.638, *P <* 0.001; Widowed, HR 1.999, 95% CI 1.829–2.185, *P <* 0.001) (Figure 1).

**Table 2:** Univariate and multivariate analysis for overall survival

Characteristic		5-year OS	Univariate analysis		Multivariate analysis		
			Log Rank	*P* value	HR	95% CI	*P* value
			χ^2^ test				
Gender							
	Male	58.1%	0.9	0.330			
	Female	57.0%					
Age							< 0.001
	< 60	64.8%	285	< 0.001	Reference		
	≥ 60	50.8%			1.617	1.526–1.713	< 0.001
Race							< 0.001
	White	59.4%	244	< 0.001	Reference		
	Black	37.3%			1.495	1.368–1.634	< 0.001
	Others	60.0%			1.125	1.003–1.263	0.045
Marital Status							< 0.001
	Married	65.6%	569	< 0.001	Reference		
	Single	49.3%			1.645	1.531–1.767	< 0.001
	Divorced/Separated	49.1%			1.517	1.404–1.638	< 0.001
	Widowed	37.5%			1.999	1.829–2.185	< 0.001
Grade							< 0.001
	I	64.2%	42.9	< 0.001	Reference		
	II	55.5%			0.990	0.906–1.081	0.819
	III	58.3%			0.803	0.729–0.884	< 0.001
	IV	62.3%			0.752	0.547–1.033	0.079
AJCC stage			596	< 0.001			< 0.001
	I	74.9%			Reference		
	II	61.3%			1.617	1.439–1.817	< 0.001
	III	57.0%			1.814	1.552–2.119	< 0.001
	IV	49.2%			1.974	1.686–2.311	< 0.001
SEER stage			1072	< 0.001			< 0.001
	Localized	70.9%			Reference		
	Regional	58.1%			1.089	0.950–1.248	0.220
	Distant	34.3%			2.004	1.722–2.332	< 0.001
Surgery							< 0.001
	Yes	62.8%	252	< 0.001	Reference		
	No	51.3%			2.416	2.010–2.904	< 0.001
Radiation							< 0.001
	Yes	52.2%	184	< 0.001	Reference		
	No	61.9%			2.238	1.864–2.687	< 0.001

Notes: OS, overall survival; HR, hazard ratio; CI, confidence interval; AJCC, the American Joint Committee on Cancer; SEER, the Surveillance, Epidemiology and End Results.

### The influence of marital status on tumor cause-specific survival (TCSS)

**Figure 2:**
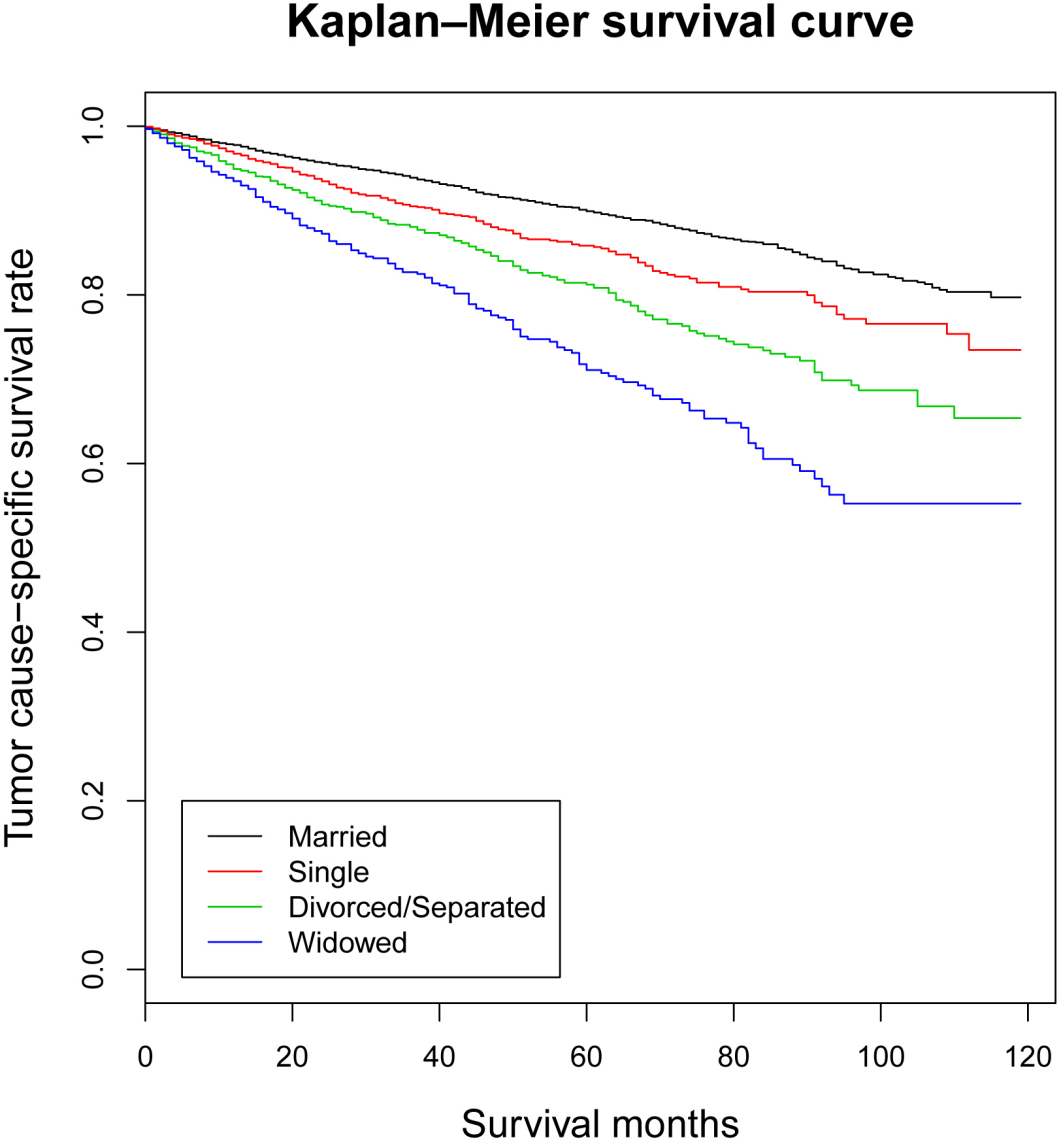
Kaplan-Meier survival curves: the tumor cause-specific survival in patients with oral tongue squamous cell carcinoma according to marital status χ^2^ = 238, *P <* 0.001.

The tumor cause-specific survival (TCSS) were also calculated through univariate analysis (Kaplan-Meier analysis) and multivariate analysis (multivariate Cox regression analysis) (Table 3 and Supplementary Table 1). The 5-year TCSS rate of the married group was 89.9%, while other 5-year TCSS rates were 85.8% in the single group, 81.2% in the divorced/separated group and 71.1% in the widowed group. Gender (*P <* 0.001), age (*P* 0.001), race (*P <* 0.001), marital status (*P <* 0.001), AJCC TNM stage (*P <* 0.001), SEER stage (*P <* 0.001), therapy of surgery (*P <* 0.001) and therapy of radiation (*P <* 0.001) were found to be associated with TCSS by univariate analysis. When the previous variables were adjusted in multivariate analysis, it revealed gender (*P* < 0.001), age (*P <* 0.001), race (*P <* 0.001), marital status (*P <* 0.001), AJCC TNM stage (*P <* 0.001) and SEER stage (*P* < 0.001) as independent prognostic factors. Moreover, as for marital status, married patients had beneficial TCSS when compared with other OTSCC patients (Single, HR 1.603, 95% CI 1.375–1.869, *P <* 0.001; Divorced/Separated, HR 1.940, 95% CI 1.675–2.247, *P <* 0.001; Widowed, HR 2.493, 95% CI 2.096–2.966, *P <* 0.001) (Figure 2).

**Table 3:** Univariate and multivariate analysis for tumor cause-specific survival

Characteristic		5-year TCSS	Univariate analysis		Multivariate analysis		
			Log Rank	*P* value	HR	95% CI	*P* value
			χ^2^ test				
Gender							< 0.001
	Male	86.5%	4.4	0.036	Reference		
	Female	87.1%			0.689	0.601–0.789	< 0.001
Age							< 0.001
	< 60	92.6%	308	< 0.001	Reference		
	≥ 60	80.3%			2.635	2.332–2.978	< 0.001
Race							< 0.001
	White	87.0%	27.6	< 0.001	Reference		
	Black	78.9%			1.450	1.193–1.762	< 0.001
	Others	89.2%			0.864	0.674–1.107	0.247
Marital Status							< 0.001
	Married	89.9%	238	< 0.001	Reference		
	Single	85.8%			1.603	1.375–1.869	< 0.001
	Divorced/Separated	81.2%			1.940	1.675–2.247	< 0.001
	Widowed	71.1%			2.493	2.096–2.966	< 0.001
Grade							
	I	87.1%	3.9	0.274			
	II	85.9%					
	III	87.6%					
	IV	84.0%					
AJCC stage			17	< 0.001			< 0.001
	I	87.9%			Reference		
	II	84.9%			1.279	1.062–1.540	0.009
	III	85.6%			1.211	0.925–1.585	0.163
	IV	87.1%			0.957	0.727–1.258	0.751
SEER stage			59.7	< 0.001			< 0.001
	Localized	87.1%			Reference		
	Regional	87.9%			0.933	0.736–1.182	0.565
	Distant	81.5%			1.664	1.255–2.206	< 0.001
Surgery			10.9	< 0.001			
	Yes	87.3%					
	No	85.9%					
Radiation							
	Yes	86.2%	6.1	0.014			
	No	87.1%					

Notes: TCSS, tumor cause-specific survival; HR, hazard ratio; CI, confidence interval; AJCC, the American Joint Committee on Cancer; SEER, the Surveillance, Epidemiology and End Results.

### Subgroup survival analysis stratified by AJCC TNM stage and SEER stage

The prognostic effect of marital status on OS and TCSS was explored in each subgroup by multivariate analysis according to AJCC TNM stage and SEER stage (Table 4). We found that marital status still acted as an independent prognostic factor for OS (*P <* 0.001) and TCSS (*P <* 0.001) in each AJCC TNM stage and SEER stage after subgroup analysis. Similarly, married patients enjoyed better results for OS and TCSS in each subgroup, while unmarried patients displayed a hazard ratio of mortality, among which the widowed patients almost exhibited with the highest risk (Supplementary Figure 1 and Supplementary Figure 2). However, we found no significant difference between the married and single group associated with OS in the subgroup of AJCC TNM stage I, as well as with TCSS in the subgroup of AJCC stage III.

**Table 4:** Multivariate analysis of marital status for overall survival and tumor cause-specific survival

Variable		Overall survival			Tumor cause-specific survival		
		HR	95% CI	*P* value	HR	95% CI	*P* value
**SEER stage**							
Localized							
	Married	Reference			Reference		
	Single	1.369	1.156–1.621	< 0.001	1.431	1.084–1.890	0.012
	Divorced/Separated	1.366	1.135–1.644	< 0.001	1.633	1.232–2.164	< 0.001
	Widowed	1.944	1.633–2.315	< 0.001	2.619	2.013–3.407	< 0.001
Regional							
	Married	Reference			Reference		
	Single	1.760	1.591–1.946	< 0.001	1.672	1.331–2.100	< 0.001
	Divorced/Separated	1.518	1.362–1.692	< 0.001	2.007	1.624–2.480	< 0.001
	Widowed	1.977	1.723–2.269	< 0.001	2.696	2.048–3.550	< 0.001
Distant							
	Married	Reference			Reference		
	Single	1.545	1.355–1.763	< 0.001	1.662	1.206–2.290	0.002
	Divorced/Separated	1.506	1.311–1.730	< 0.001	2.139	1.578–2.900	< 0.001
	Widowed	1.710	1.420–2.060	< 0.001	1.680	1.078–2.618	0.022
**AJCC stage**							
I							
	Married	Reference			Reference		
	Single	1.207	0.964–1.512	0.101	1.434	1.020–2.017	0.038
	Divorced/Separated	1.455	1.151–1.841	0.002	1.641	1.157–2.327	0.005
	Widowed	1.778	1.422–2.222	< 0.001	2.555	1.845–3.538	< 0.001
II							
	Married	Reference			Reference		
	Single	1.778	1.423–2.221	< 0.001	2.045	1.371–3.051	< 0.001
	Divorced/Separated	1.408	1.107–1.792	0.005	2.112	1.454–3.069	< 0.001
	Widowed	2.014	1.594–2.544	< 0.001	2.383	1.567–3.623	< 0.001
III							
	Married	Reference			Reference		
	Single	1.747	1.475–2.070	< 0.001	1.409	0.986–2.014	0.060
	Divorced/Separated	1.534	1.268–1.856	< 0.001	1.756	1.244–2.480	0.001
	Widowed	1.868	1.511–2.309	< 0.001	1.959	1.308–2.936	0.001
IV							
	Married	Reference			Reference		
	Single	1.666	1.519–1.827	< 0.001	1.629	1.298–2.044	< 0.001
	Divorced/Separated	1.541	1.397–1.700	< 0.001	2.073	1.675–2.564	< 0.001
	Widowed	1.976	1.733–2.253	< 0.001	2.686	2.000–3.607	< 0.001

Notes: HR, hazard ratio; CI, confidence interval; SEER, the Surveillance, Epidemiology and End Results; AJCC, the American Joint Committee on Cancer.

## DISCUSSION

In this study, we firstly explored the influence of marital status on overall survival and tumor cause-specific survival in patients with oral tongue squamous cell carcinoma. As a result, we found that the married patients experienced better overall survival and tumor cause-specific survival than the single, divorced/separated, widowed patients. It was discovered that married patients had beneficial survival results in oral tongue squamous cell carcinoma significantly, which remained even after adjusted for age, race, grade, AJCC TNM stage, SEER stage, therapy of surgery and therapy of radiation in multivariable analyses. Subgroup analysis confirmed the conservatory role of marriage based on different AJCC TNM stage and SEER stage. In addition, it pointed out that the widowed patients always endured the highest risk of mortality for OS and TCSS. The meaning of this study lies in the important impact of marital status on survival of oral tongue squamous cell carcinoma, which is in consistency with previous researches of other cancers [17–20].

Engel put forward a novel conception of biopsychosocial medical model replacing biomedical model in 1977 [7]. It attributed occurrence, development and outcome of diseases to biological factors like genetic element, psychological factors like mood or behavior element, and social factors like or familial or cultural element [21]. Since then, plenty of studies have been performed to dig out the relation between biopsychosocial factors and diverse diseases, such as ischemic heart disease, diabetes mellitus and chronic pain [22–24]. Gradually, importance has been attached to the function of biopsychosocial factors in cancer patients [25, 26]. A longitudinal study aiming at marital status and mortality in British women found that being single was associated with higher mortality instead of being divorced and being widowed [27]. Another research analyzed marital status and head and neck cancer outcomes based on 51,272 patients from SEER database, found that the married patients were less likely to present with metastatic disease, while the married patients were more likely to receive definitive treatment [17]. A cohort study in Swedish discovered that divorce, widowhood, living alone, low educational attainment, and low income increased the risk of subtypes in esophageal and gastric cancer [28]. Identically, our study found out that marital status of married patients played a beneficial role in survival outcomes of oral tongue squamous cell carcinoma patients, which was in accordance with the previous researches. Whereas, it emphasized the interrelated relationship between marital status and survival rather than the causal relationship. It is essential to dig out the latent mechanism how marital status influences survival outcomes in order to improve the outcome of tongue cancer patients [29].

Our result showed that marital status was found to be associated with survival in patients with oral tongue squamous cell carcinoma, but why marital status of married patients served as a protective factor? Firstly, a beatific marriage brought a comfortable, confident and enjoyable emotional state. So the married group of cancer patients could receive social support from friends and family, which would decrease their risk for psychological distress. Single patients were found to have high rates of distress [30]. Patients with depression symptoms suffered more during cancer treatment, and it was verified that less social support were associated with worse mortality [31]. The molecular mechanism involved several inflammatory biomarkers such as IL-1β, IL-6, TNF and C-reactive protein [32]. Secondly, stable marriage in married patients always came with good family financial circumstances. And the financial circumstances was associated with cancer survival, mainly explained by stage at diagnosis and differences in treatment [33]. Those with fine financial states were likely to obtain early medical examination and were consequently detected with early tumor stage. They could also receive early and better treatment [34]. Thirdly, compared with unmarried patients, married patients were linked with more nodes through more connection in the social network. And they may get more information about medical facilities, professional experts and treatments, contributing to a better prognosis [35]. Nowadays the complex cancer therapy made it difficult for the unmarried patients to follow up [36]. The social network impacted the patient’s adherence, and a long-term adherence can affect patients’ health outcomes [37]. Furthermore, low social network diversity was independently associated with more adverse lifestyle associated with prognosis [38].

Although we included a large number of sample size in this study, there existed several limitations interfering the results. Firstly, the information of marital status provided by the SEER database is not complete enough. It only offers marital status at diagnosis but lacks changes during the follow-up period. However, the alteration of marital status during the follow-up period probably affected the survival outcomes, since more than one half of patients with oral tongue squamous cell carcinoma would survive for five years. In addition, the patients prevalently aged more than 60 years old, implying the potential of transformation from married to widowed during the follow-up period. Secondly, besides marital status, there are many other social factors such as education, income and insurance included in the biopsychosocial medical model. While the SEER database are short of information about those other social factors, confusing the survival outcomes in the married patients. We had better take all these factors into account if possible. Thirdly, the contentment degree of marriage is unreachable in the SEER database. But even patients from the same married group own different marital satisfaction. For example, marital distress has negative health consequences over time through damaging immune system. So it is unavailable to explore relation between quantized marital status and survival [39]. Fourthly, it lacks more detailed clinical records like surgery and radiation information since diagnosis, which may contribute to bias. Fifthly, the SEER database did not take into account patients with sexual minority preferences including lesbian, gay, bisexual and transgender peoples. However, the factors of sexual orientation have been demonstrated to be associated with cancer survival by several studies [40–42]. As for subgroup analysis the size of each subgroup was relatively small. More studies with thorough information and larger sample size in the future are necessary to prove our results.

Besides, there are a number of well-known inherent limitations in the SEER database. On one hand, some measures are problematic. For example, behavioral and other patient risk factors such as smoking or alcohol use, body mass index (BMI) and personal or family history are incomplete, which may affect the cancer survival. Additionally, information about recurrence is seldom collected by any SEER registry [43, 44]. On the other hand, some important measures can’t be achieved from the SEER database. Firstly, there is no patient self-reported information including functional status or patient’s quality of life (QoL) in the SEER data, and the information reflect patients’ survival quality. Secondly, the SEER data only record the vital status during follow-up. Neither information of metastasis occurring after initial diagnosis nor site of metastasis is provided [45, 46]. Thirdly, test results from lab tests and imaging are not supplied in the SEER database. So the data of tumor marker associated with cancer survival are missing for analysis further.

In spite of those limitations mentioned above, we affirmed the beneficial survival results of married patients in oral tongue squamous cell carcinoma. On the contrary, unmarried patients suffered from high risk of overall and tumor cause-specific mortality. Particularly the widowed patients always endured the highest risk of mortality among the unmarried patients. According to the biopsychosocial medical model, it is the underlying psychological and social support that produces a protective power, improving the final survival outcomes [47, 48]. So, when it comes to healthcare providers, these patients faced with higher risk of mortality demand more elaborate care in clinical practice, to strengthen the psychosocial support and construct their social network.

## MATERIALS AND METHODS

### Data sources

The open dataset was obtained from the Surveillance, Epidemiology, and End Results database released in November 2015 through internet access (https://seer.cancer.gov). It included demographic information like age, sex, race, marital status, and clinical records of stage, grade, therapy, as well as follow-up data. We took the SEER November 2015 Research Data for analyses, which contained the SEER 18 registries Research Data and the Hurricane Katrina Impacted Louisiana Cases from 1973 to 2013.

### Inclusion and exclusion criteria

We extracted patients with oral tongue squamous cell carcinoma (International Classification of Diseases for Oncology, Third Edition [ICD-O-3], code C01.9, C02.0, C02.1, C02.2, C02.3, C02.4, C02.8, C02.9) for our study. Patients were included when meeting the following criteria: (1) patients were aged 18 years or older at diagnosis; (2) oral tongue carcinoma was diagnosed between 2004 and 2013; (3) histological types were limited to squamous cell carcinoma (code 8050, 8051, 8052, 8070, 8071, 8072, 8073, 8074, 8075, 8076, 8081, 8082, 8083 and 8084). Patients were excluded according to the following criteria: (1) age at diagnosis was less than 18 years; (2) incomplete clinical information; (3) unknown demographic information; (4) unknown cause of death or unknown survival month.

### Statistical analysis

Data of gender, age, race, marital status, grade, AJCC TNM stage, SEER stage, therapy, cause of death and survival months were collected from the SEER database. We described continuous variables as means and standard deviations, while described categorical variables as frequencies and percentages. For categorical variables, we chose the Pearson’s chi-squared test and Fisher’s exact tests to detect the statistical difference. For continuous variables, we chose independent Student’s *t*-test and Analysis of Variance (ANOVA). When homogeneity of variance did not correspond, nonparametric test of Kruskal-Wallis test was adopted. Besides, we selected Kaplan-Meier analysis and multivariate Cox regression models to distinguish risk factors for overall survival (OS) and tumor cause-specific survival (TCSS). For overall survival analysis, any cause of deaths was defined as events and sur*vivo*rs were defined as censored events. For tumor cause-specific survival, deaths caused by tongue cancer were considered as events and deaths by other causes or sur*vivo*rs were considered as censored events. All the data analysis in this study was conducted by R statistical software version 3.3 (https://www.r-project.org). All *P* values were two-sided and *P <* 0.05 was considered significant.

## SUPPLEMENTARY MATERIALS FIGURES AND TABLE



## Abbreviations

OTSCC: Oral tongue squamous cell carcinoma
OCSCC: oral cavity squamous cell carcinoma
HPV: Human papilloma virus
TNM: Tumor node metastases
AJCC: The American Joint Committee on Cancer
SEER: The Surveillance, Epidemiology, and End Results
OS: Overall survival
TCSS: Tumor cause-specific survival
HR: Hazard ratio
CI: Confidence interval
ICD-O-3: International Classification of Diseases for Oncology, Third Edition
