# Supplementary material for: The influence of marital status on survival in patients with oral tongue squamous cell carcinoma
Source: Oncotarget. 2017 Jun 17;8(47):82092–102. doi: 10.18632/oncotarget.18538 (PMC5669873; doi:10.18632/oncotarget.18538)
Supplement: Supplementary file 1 [file oncotarget-08-82092-s001.pdf]

The influence of marital status on survival in patients with oral tongue squamous cell carcinoma

Supplementary Materials

Supplementary Table 1: Pairwise comparison of univariate analysis among married, single, divorced/separated and widowed group

Characteristic	Overall survival		Tumor cause-specific survival	
	Log Rank χ^2 test	*P* value	Log Rank χ^2 test	*P* value
Married-Single	239	< 0.001	23.1	< 0.001
Married-Divorced/Separated	196	< 0.001	92.6	< 0.001
Married-Widowed	416	< 0.001	204	< 0.001
Single-Divorced/Separated	0	0.943	13.9	< 0.001
Single-Widowed	43.5	< 0.001	61.9	< 0.001
Divorced/Separated-Widowed	40.3	< 0.001	18.4	< 0.001

Notes: OS, overall survival; TCSS, tumor cause-specific survival.

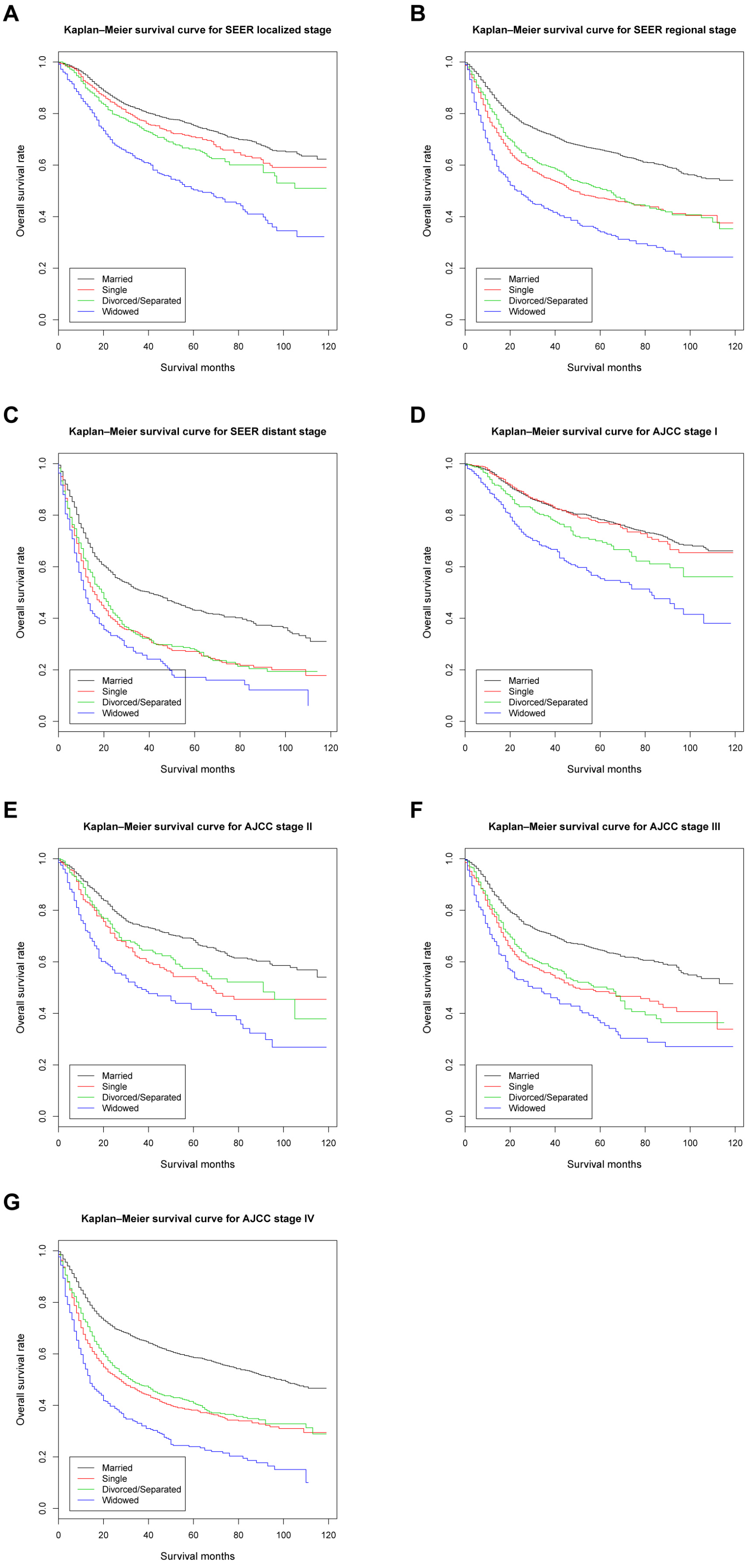

Supplementary Figure 1: Kaplan-Meier survival curves: the overall survival in patients with oral tongue squamous cell carcinoma according to marital status. (**A**) SEER localized stage: $\chi^2 = 133$, $P < 0.001$; (**B**) SEER regional stage: $\chi^2 = 334$, $P < 0.001$; (**C**) SEER distant stage: $\chi^2 = 106$, $P < 0.001$; (**D**) AJCC stage I: $\chi^2 = 77.5$, $P < 0.001$; (**E**) AJCC stage II: $\chi^2 = 65.8$, $P < 0.001$; (**F**) AJCC stage III: $\chi^2 = 94.7$, $P < 0.001$; (**G**) AJCC stage I: $\chi^2 = 400$, $P < 0.001$.

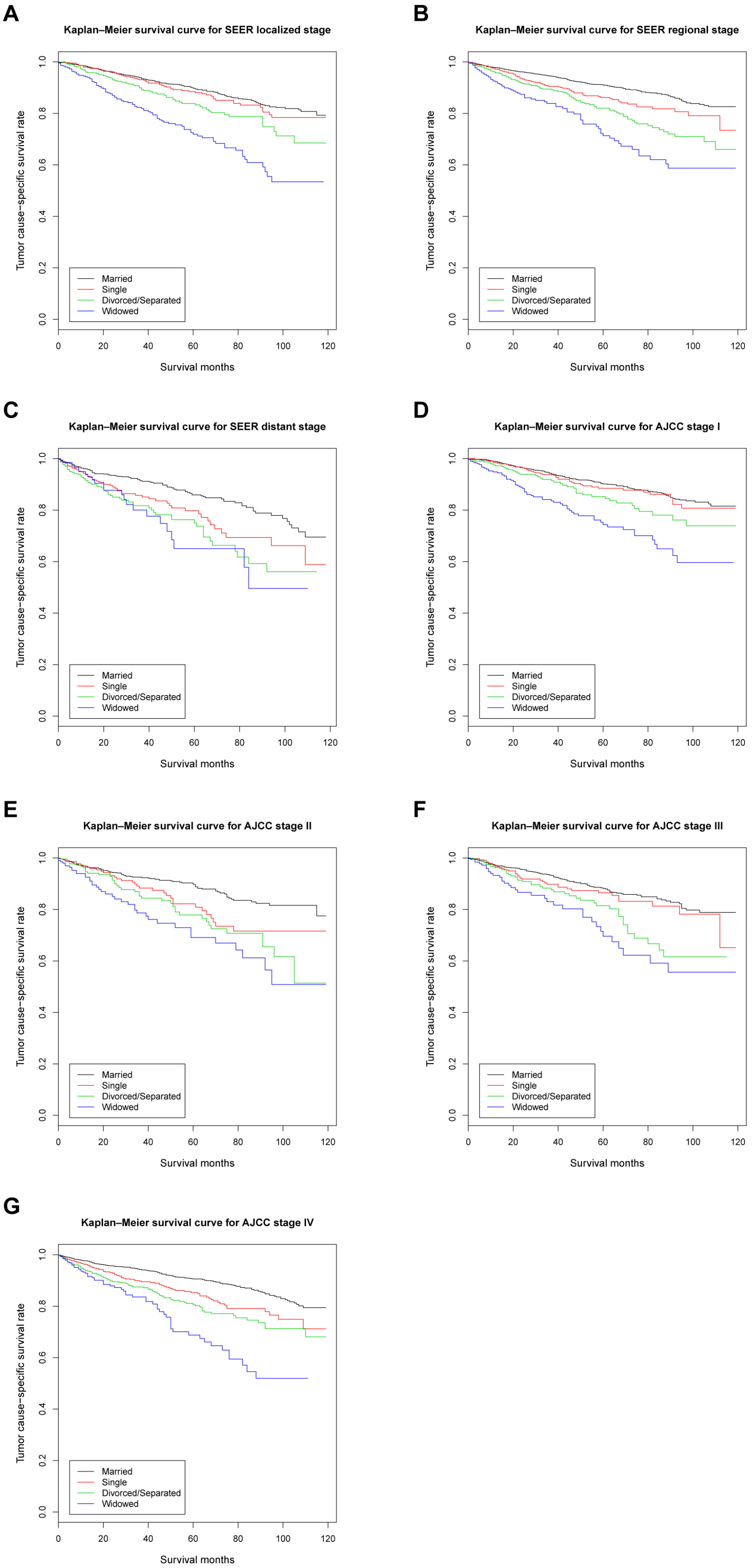

Supplementary Figure 2: Kaplan-Meier survival curves: the tumor cause-specific survival in patients with oral tongue squamous cell carcinoma according to marital status. (**A**) SEER localized stage: $\chi^2 = 97.4$, $P < 0.001$; (**B**) SEER regional stage: $\chi^2 = 118$, $P < 0.001$; (**C**) SEER distant stage: $\chi^2 = 30.1$, $P < 0.001$; (**D**) AJCC stage I: $\chi^2 = 58.4$, $P < 0.001$; (**E**) AJCC stage II: $\chi^2 = 38.9$, $P < 0.001$; (**F**) AJCC stage III: $\chi^2 = 35.2$, $P < 0.001$; (**G**) AJCC stage I: $\chi^2 = 108$, $P < 0.001$.
